# MiR-874-3p represses the migration and invasion yet promotes the apoptosis and cisplatin sensitivity via being sponged by long intergenic non-coding RNA 00922 (LINC00922) and targeting Glycerophosphodiester Phosphodiesterase Domain Containing 5 (GDPD5) in gastric cancer cells

**DOI:** 10.1080/21655979.2022.2045831

**Published:** 2022-03-12

**Authors:** Xiaoyu Zhang, Xudong Dai, Xin Zhao, Jian Wang, Jin Dou, Haiwen Zhuang, Ning Chen, Haijian Zhao

**Affiliations:** a Division of Gastrointestinal Surgery, Department of General Surgery, The Affiliated Huai’an Hospital of Xuzhou Medical University, Huaian, Jiangsu, China; b Department of General Surgery, Lianshui People’s Hospital Affiliated to Kangda College of Nanjing Medical University, Huaian, Jiangsu, China

**Keywords:** MicroRNA-874-3p, gastric cancer, long intergenic non-coding RNA 00922, cisplatin, Glycerophosphodiester Phosphodiesterase Domain Containing 5

## Abstract

Our study mainly reports the specific mechanisms of microRNA (miR)-874-3p on drug resistance in gastric cancer (GC). Clinical specimen was collected. The upstream long non-coding RNA (lncRNA) and the downstream gene of miR-874-3p were predicted using bioinformatic analysis with the results being ascertained with dual-luciferase reporter assay. The viability, apoptosis, migration and invasion of transfected GC cells with or without cisplatin (DDP) treatment were evaluated by Cell Counting Kit-8 (CCK-8), flow cytometric, Scratch, and Transwell assays. An animal xenograft model was constructed. Expressions of long intergenic non-coding RNA 00922 (LINC00922), miR-874-3p and potential target genes were quantified by quantitative real-time polymerase-chain reaction (qRT-PCR) and Western blot. MiR-874-3p, which was lower-expressed in drug-resistant GC tissues and cells, was upregulated to repress the viability, migration and invasion but enhance the apoptosis and sensitivity in GC cells with or without DDP resistance. Downregulation of miR-874-3p eliminated the effects of silenced LINC00922, a upstream lncRNA of miR-874-3p, on cell viability, apoptosis, migration and invasion, as well as the expressions of Glycerophosphodiester Phosphodiesterase Domain Containing 5 (GDPD5) and the downstream gene of miR-874-3p in DDP-resistant GC cells. GDPD5 silencing diminished the effects of miR-874-3p downregulation on GDPD5 expression, viability, migration and invasion of DDP-resistant GC cells. Additionally, LINC00922 silencing enhanced the inhibitory effect of DDP on tumor growth, whereas reversing the effects of DDP on LINC00922, miR-874-3p and GDPD5 expressions in tumors. MiR-874-3p, an miRNA, which is sponged by LINC00922 and targets GDPD5, inhibits the GC progression yet enhances the DDP sensitivity in GC.

## Graphical abstract

**Figure:**
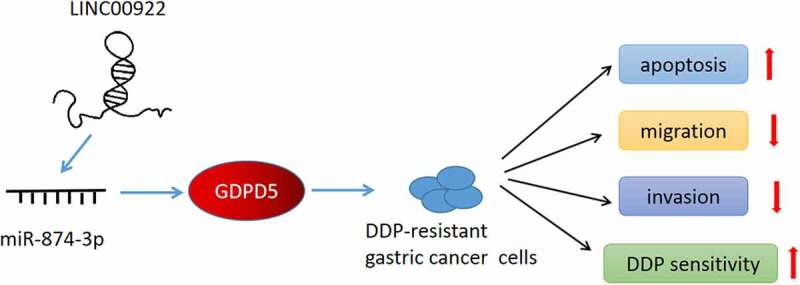

## Introduction

As a molecularly and phenotypically highly heterogeneous disease, at present, gastric cancer (GC) is the fifth most prevalent malignancy and ranks the third in the most common cause of cancer-associated death. What’s worse, most patients with GC are diagnosed at an advanced stage where the treatment is often proved to be ineffective [1,2]. Currently, cisplatin (DDP)-based therapy is frequently applied for the treatment in patients with advanced GC. However, DDP-associated drug resistance and DDP-induced adverse effects in patients limit its clinical efficacy, making it urgent to further detect and determine the detailed molecular mechanism concerning the drug resistance in GC so as to work out the potential therapeutic strategies for GC [3–5].

Prior discoveries have underlined the different yet peculiar roles of non-coding RNAs (ncRNAs) in both the development, progression, and the participation of drug-resistance in GC, in which microRNAs (miRNAs, miRs) have been suggested to play critical roles [6–8]. It has been emphasized that suppression on miR-198 is implicated in the mechanism via which circRNA AKT3 (circ_AKT3) enhances DDP resistance in GC, for example [9]. MiR-374a-5p has been recently unveiled as a novel therapeutic target for both diagnosis and drug resistance therapy in GC [10]. Besides, miR-31 possesses the inhibitory effects on both RhoA-mediated invasion and resistance against chemotherapy in GC cell line MKN-45 [11]. Apart from the discoveries above, previous reports have identified miR-874-3p as a novel miRNA that participates in several pathological processes, such as cancer, myocardial infraction, osteogenesis and erectile dysfunction, osteoporosis [12,13]. When it comes to GC, previous reports have unveiled the inhibitory effects of miR-874-3p on the proliferation, migration, and invasion as well as the metastasis-associated traits of GC cells and its regulatory effects on drug resistance in GC [14–16]. Furthermore, it has also been suggested that miRNAs could be ‘sponged’ by long non-coding RNAs (lncRNAs), another member of the ncRNA family that has been uncovered as the biomarkers to be implicated in both the progression and drug resistance of GC as well [17–19]. Nevertheless, there was inadequate discussion concerning the interaction between miR-874-3p and lncRNAs in the progression and drug resistance of GC.

In our current study, we aimed to reevaluate the effects of miR-874-3p on GC and try to identify the mechanism via which miR-874-3p elicited its effects on GC, hoping to figure out a possibly viable therapy for GC in clinical practice.

## Materials and methods

### Ethical statement

Our current study has been approved by the Ethics Committee of Huai’an Second People’s Hospital, The Affiliated Huai’an Hospital of Xuzhou Medical University (endorse number: HEYLL201958). Prior to the initiation of our study, all patients volunteering to participate in our study have signed the written informed consent.

Our current study has also obtained the approval by the Ethics Committee of Experimental Animals of Nanfang Hospital (endorse number: XHNK-2018012501-2). All animal experiments were performed in Nanfang Hospital strictly following the guidelines of China Council on Animal Care and Use. Every effort in the experiment was made for the minimization of pain and discomfort to the animals.

### Data acquisition and bioinformatic analysis

To confirm the upstream lncRNA of miR-874-3p, LncBase Predicted V2 (http://carolina.imis.athena-innovation.gr/diana_tools) was used to predict the complementary-binding sites. Besides, to predict the downstream target genes of miR-874-3p, several other databases were employed, including TargetScan V7.2 (www.targetscan.org/vert_72), StarBase (starbase.sysu.edu.cn), and The Cancer Genome Atlas Stomach Adenocarcinoma (TCGA-STAD, www.genome.gov/Funded-Programs-Projects/Cancer-Genome-Atlas). The results were finally summarized using a Venn diagram which was drawn and downloaded from Venny online software (v. 2.1.0, bioinfogp.cnb.csic.es/tools/venny). The specific results can be seen in Figure 6(a) of our study.

**Figure 1.**
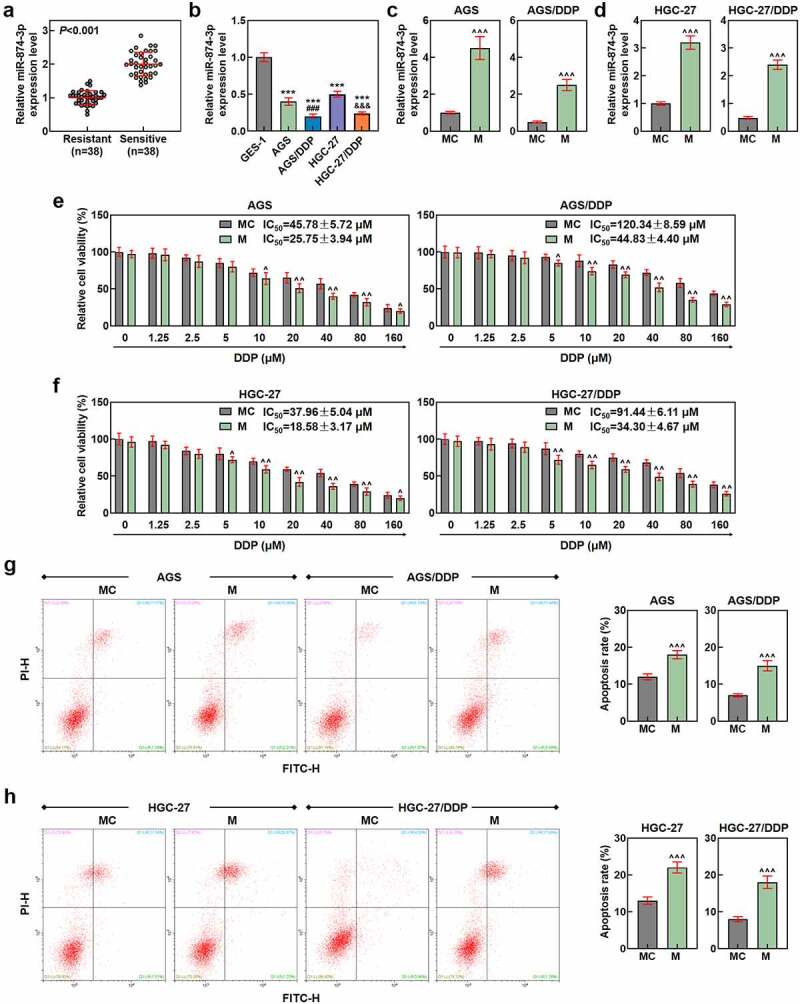
MiR-874-3p was lower-expressed in drug-resistant GC, and miR-874-3p upregulation repressed the cell viability but enhanced sensitivity and apoptosis in GC cells with or without DDP. (a) Relative miR-874-3p expression in drug-resistant (Resistant) and drug-sensitive (Sensitive) GC tissues was quantified using qRT-PCR. U6 was employed as the internal control. (b) Relative miR-874-3p expression in DDP-resistant or parental GC cell and gastric mucosal epithelial cell line GES-1 was measured with qRT-PCR. U6 was applied as the internal control. (c-d) Relative miR-874-3p expression in DDP-resistant or parental GC cell AGS (c) and HGC-27 (d) after transfection was quantified with qRT-PCR. U6 was the internal control. (e-f) The effects of miR-874-3p on the viability of DDP-resistant or parental GC cell AGS (e) and HGC-27 (f) after the administration of DDP (0, 1.25, 2.5, 5, 10, 20, 40, 80 and 160 μmol/L) for 48 hours were assessed with CCK-8 assay. (g-h) The effects of miR-874-3p on the apoptosis of GC cell AGS (g) and HGC-27 (h) with or without 20 μmol/L DDP treatment for 48 hours were confirmed with flow cytometric assay. All data were expressed as mean ± standard deviation (SD), which was indicative of three independent tests. ***p < 0.001, vs. GES-1; ^###^*p < *0.001, vs. AGS; ^&&&^*p < *0.001, vs. HGC-27; ^^^*p < *0.05, ^^^^*p < *0.01, ^^^^^*p < *0.001, vs. MC. miR: microRNA; GC: gastric cancer; DDP: cisplatin; qRT-PCR: quantitative real-time polymerase chain reaction; IC_50_: the half maximal inhibitory concentration; MC: mimic control; M: mimic; CCK-8: Cell Counting Kit-8.

**Figure 2.**
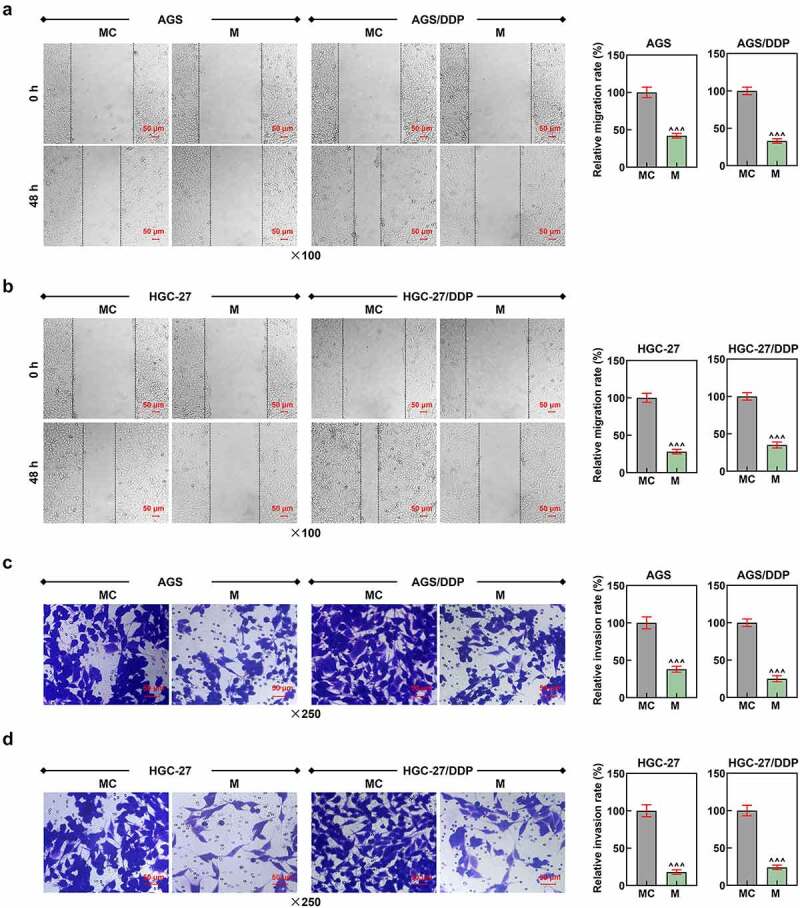
MiR-874-3p upregulation suppressed the migration and invasion in GC cells with or without DDP. (a-b) The effects of miR-874-3p on the migration of DDP-resistant or parental GC cell AGS (a) and HGC-27 (b)  were confirmed with Scratch assay at 0 and 48 hours under × 100 magnification (Scale bar = 50 μm). (c-d) The effects of miR-874-3p on the invasion of DDP-resistant or parental GC cell AGS (c) and HGC-27 (d)  were unveiled with Transwell assay at 48 hours (× 250 magnification; Scale bar = 50 μm). All data were expressed as mean ± standard deviation (SD), which was indicative of three independent tests. ^^^^^*p < *0.001, vs. MC.

**Figure 3.**
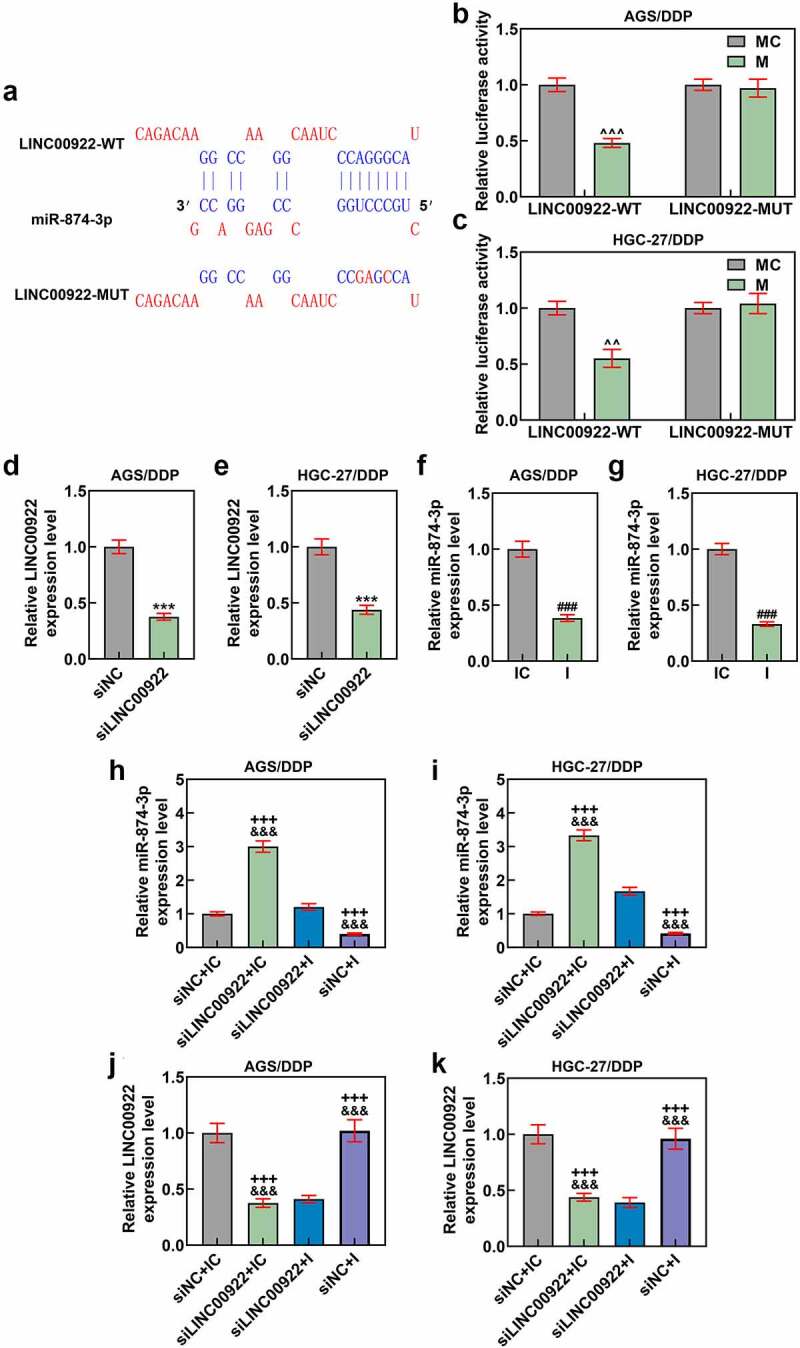
LINC00922 was the upstream lncRNA for miR-874-3p, and miR-874-3p downregulation eliminated the effects of LINC00922 silencing on miR-874-3p expression in DDP-resistant GC cells. (a-c) LncBase Predicted V2 (http://carolina.imis.athena-innovation.gr/diana_tools) (a) predicted and dual-luciferase reporter assay (b-c) confirmed LINC00922 as the upstream lncRNA for miR-874-3p. (d-g) Relative expressions of LINC00922 (d-e) and miR-874-3p (f-g) in DDP-resistant GC cell AGS and HGC-27 after transfection were quantified with qRT-PCR. GAPDH (for LINC00922) and U6 (for miR-874-3p) were the internal controls. (h-i) Relative miR-874-3p expression in DDP-resistant GC cell AGS (h) and HGC-27 (i) after transfection was quantified with qRT-PCR. (j-k) Relative LINC00922 expression in DDP-resistant GC cell AGS/DDP (j) and HGC-27/DDP (k) after transfection was quantified with qRT-PCR. U6 was used as the internal control. All data were expressed as mean ± standard deviation (SD), which was indicative of three independent tests. ^^^^*p < *0.01, ^^^^^*p < *0.001, vs. MC; ***p < 0.001, vs. siNC; ^###^*p < *0.001, vs. IC; ^+++^*p < *0.001, vs. siNC+IC; ^&&&^*p < *0.001, vs. siLINC00922 + I. LINC00922: long intergenic non-coding RNA 00922; LncRNA: long non-coding RNA; WT: wild-type; MUT: mutated; siRNA: small interfering RNA; IC: inhibitor control; I: inhibitor.

**Figure 4.**
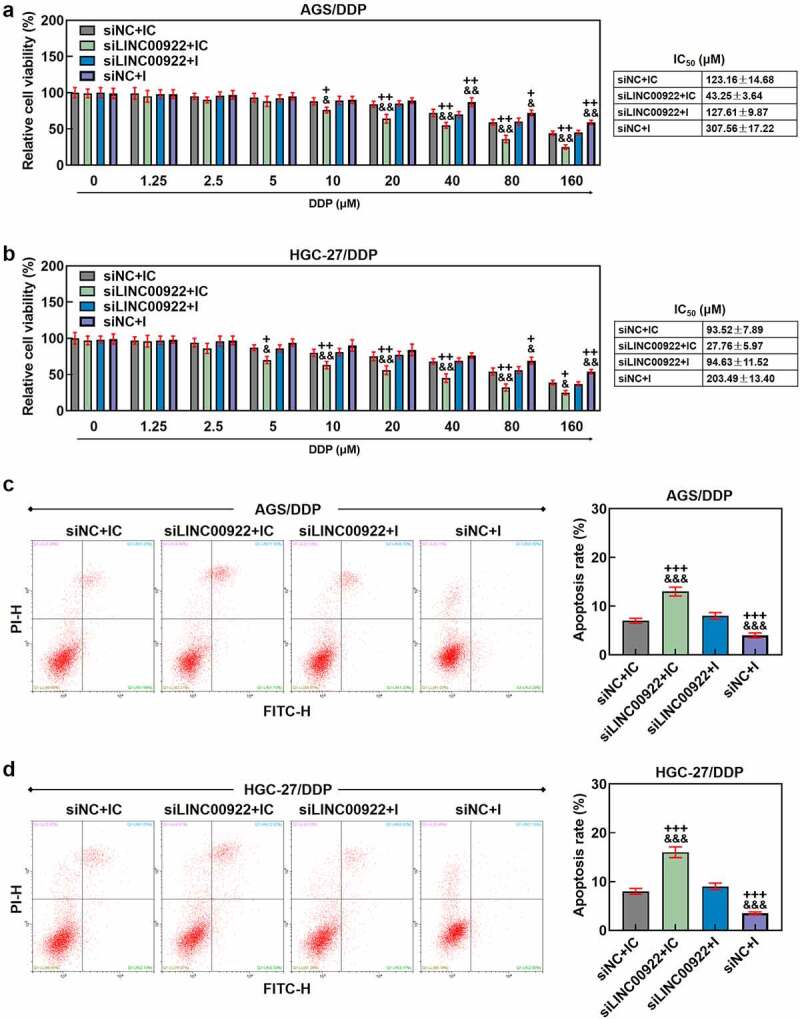
MiR-874-3p downregulation eliminated the effects of LINC00922 silencing on cell viability and apoptosis in DDP-resistant GC cells (a-b) The effects of LINC00922 silencing and miR-874-3p downregulation on the viability in DDP-resistant GC cell AGS (a) and HGC-27 (b) after the administration of DDP (0, 1.25, 2.5, 5, 10, 20, 40, 80 and 160 μmol/L) for 48 hours were determined with CCK-8 assay. (c-d) The effects of LINC00922 silencing and miR-874-3p downregulation on the apoptosis of DDP-resistant GC cell AGS (c) and HGC-27 (d) were evaluated with flow cytometry. All data were expressed as mean ± standard deviation (SD), which was indicative of three independent tests. ^+^*p < *0.05, ^++^*p < *0.01, ^+++^*p < *0.001, vs. siNC+IC; ^&^*p < *0.05, ^&&^*p < *0.01, ^&&&^*p < *0.001, vs. siLINC00922 + I.

**Figure 5.**
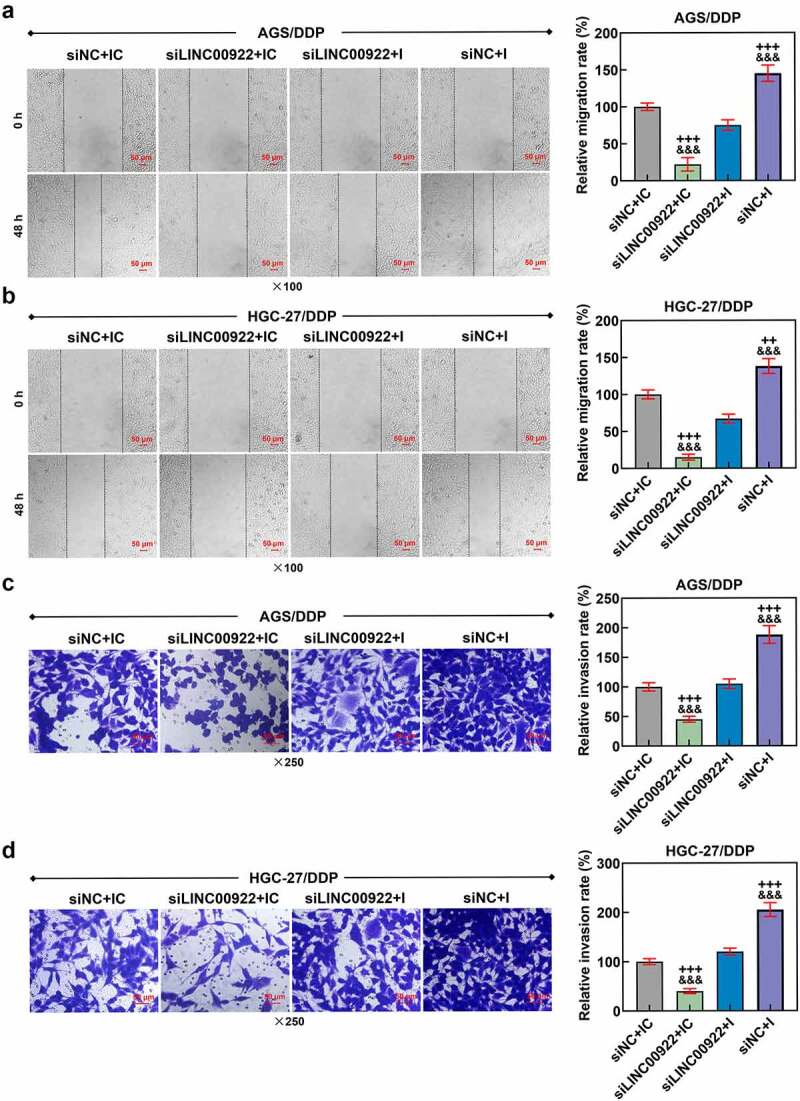
Downregulated miR-874-3p eliminated the effects of LINC00922 silencing on cell migration and invasion in DDP-resistant GC cells. (a-b) The effects of LINC00922 silencing and miR-874-3p downregulation on the migration of DDP-resistant GC cell AGS (a) and HGC-27 (b) were confirmed with Scratch assay at 0 and 48 hours, under × 100 magnification (Scale bar = 50 μm). (c-d) The effects of LINC00922 silencing and miR-874-3p downregulation on the invasion of DDP-resistant GC cell AGS (c) and HGC-27 (d) at 48 hours were detected with Transwell assay (× 250 magnification; Scale bar = 50 μm). All data were expressed as mean ± standard deviation (SD), which was indicative of three independent tests. ^+++^*p < *0.001, vs. siNC+IC; ^&&&^*p < *0.001, vs. siLINC00922 + I.

**Figure 6.**
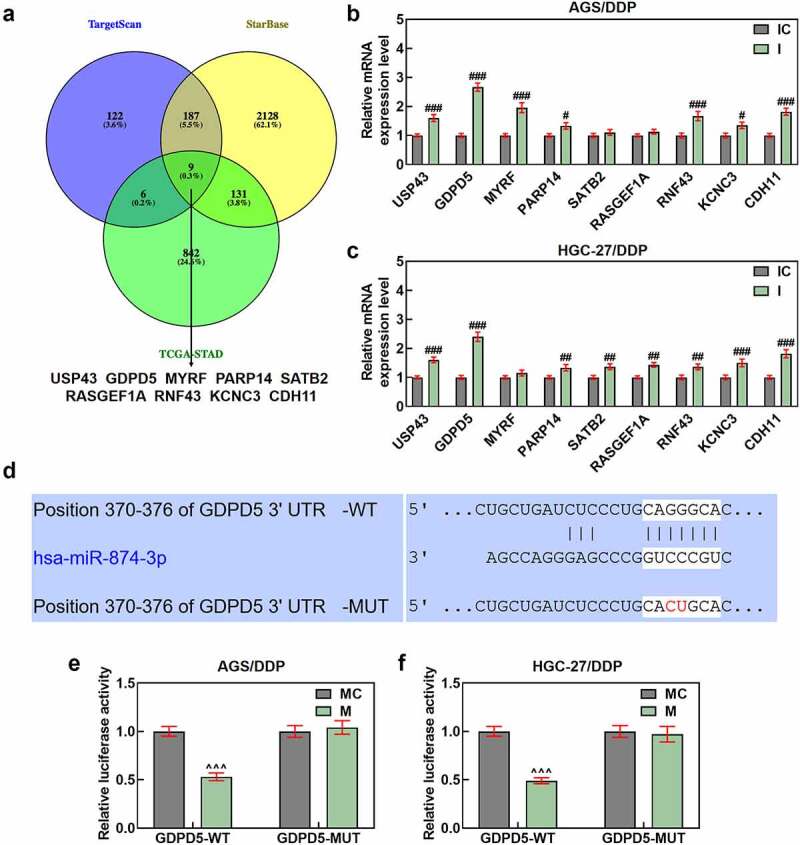
GDPD5 was the target gene of miR-874-3p. (a) The downstream target gene of miR-874-3p was predicted and confirmed as well, and the databases, including TargetScan (www.targetscan.org/vert_72), StarBase (starbase.sysu.edu.cn), and TCGA-STAD, www.genome.gov/Funded-Programs-Projects/Cancer-Genome-Atlas) were utilized. The results concerning the possible downstream target genes were finally sorted and presented using a Venn diagram **(A)**. (b-c) The expression of the potential target genes of miR-874-3p after downregulating miR-874-3p was measured in DDP-resistant GC cell AGS (b) and HGC-27 (c) by qRT-PCR. GAPDH was used as the housekeeping gene. (d-f) Data from TargetScan (d) and dual-luciferase reporter assay (e-f) confirmed GDPD5 as the target gene of miR-874-3p. All data were expressed as mean ± standard deviation (SD), which was indicative of three independent tests. ^#^*p < *0.05, ^##^*p < *0.01, ^###^*p < *0.001, vs. IC; ^^^^^*p < *0.001, vs. MC. TCGA-STAD: The Cancer Genome Atlas Stomach Adenocarcinoma; USP43: ubiquitin specific peptidase 43; GDPD5: Glycerophosphodiester Phosphodiesterase Domain Containing 5; MYRF: myelin regulatory factor; PARP14: poly(ADP-ribose) polymerase family member 14; SATB2: SATB homeobox 2; RASGEF1A: RasGEF domain family member 1A; KCNC3: potassium voltage-gated channel subfamily C member 3; CDH11: cadherin 11.

### Clinical specimen collection

A total number of 76 GC tissues, including drug-resistant GC tissue (Resistant, n = 38) and drug-sensitive GC tissue (Sensitive, n = 38), were collected from patients diagnosed with GC in Huai’an Second People’s Hospital, The Affiliated Huai’an Hospital of Xuzhou Medical University between 2018 and 2019 January. The criteria for judging cisplatin-resistant and sensitive patients were followed by standard CDDP response definitions published elsewhere [20]. All tissues were available during the resection or biopsy, and snap frozen in liquid nitrogen following the washes with saline (ST341, Beyotime Biotech, Shanghai, China).

### Cell culture and drug-resistant cell line construction

Human gastric mucosal epithelial cell line GES-1 (iCell-h062) as well as GC cell lines AGS (iCell-h016) and HGC-27 (iCell-h095) were bought from iCell (Shanghai, China, www.icellbioscience.com). Then, GES-1 cells were cultured in Roswell Park Memorial Institute-1640 (RPMI-1640) medium (11879–020, Gibco, Waltham, MA, USA) blended with 10% fetal bovine serum (FBS, 16140–063, Gibco, USA) and 1% antibiotics (penicillin-streptomycin, 15070–063, Gibco, USA). In terms of AGS cells, Ham’s F-12 K medium (21227–022, Gibco, USA) was applied for the culture, which was supplemented with both 10% FBS and 100 U/mL antibiotics. Additionally, Dulbecco’s modified Eagle’s medium (DMEM, 11966–025, Gibco, USA) was exploited for the culture of HGC-27 cell with 10% FBS and 1% antibiotics (penicillin-streptomycin). All cells were maintained in Heracell™ VIOS 160i CO_2_ incubator (51033556, ThermoFisher Scientific, Waltham, MA, USA) at 37°C with 5% CO_2_.

All procedures concerning the drug-resistant cell line construction on both AGS and HGC-27 cells were confirmed in line with a prior publication [21]. Prior to our study, DDP (IC0440) was ordered from Solarbio Life sciences (Beijing, China), and gradually added into AGS and HGC-27 cells in the logarithmic phase from 1/50 of the half maximal inhibitory concentration (IC_50_) for over 6 months. To maintain resistance to DDP, all cells were inoculated in a RPMI-1640 medium and named as AGS/DDP and HGC-27/DDP cells for subsequent studies.

### Transfection

Before the initiation of transfection, we ordered miR-874-3p mimic (miR10004911-1-5), inhibitor (miR20004911-1-5) and their controls (mimic control, miR1N0000001-1-5; inhibitor control, miR2N0000001-1-5) from RiboBio (Guangzhou, China). According to the results from bioinformatic analysis in our study, long intergenic non-coding RNA 00922 (LINC00922) was confirmed as the upstream lncRNA for miR-874-3p, while Glycerophosphodiester Phosphodiesterase Domain Containing 5 (GDPD5) was suggested as the downstream target gene of miR-874-3p. Thus, we also purchased the small interfering RNA (siRNA) and short hairpin RNA (shRNA) for LINC00922 (siLINC00922 and shLINC00922), as well as the siRNA targeting GDPD5 (siGDPD5) and their negative control (NC) from GenePharma (Shanghai, China). All sequences were available in Table 1.

**Table 1.** Sequences for transfection

Gene	Sequence (5’->-3’)
miR-874-3p mimic	CUGCCCUGGCCCGAGGGACCGA
miR-874-3p mimic control	CUGAGGGUGCCCACCGCCCGAG
miR-874-3p inhibitor	UCGGUCCCUCGGGCCAGGGCAG
miR-874-3p inhibitor control	CUCGGGCGGUGGGCACCCUCAG
siLINC00922 sense obligo	UAUUCAUGAAAAAUAGGACAG
siLINC00922 antisense obligo	GUCCUAUUUUUCAUGAAUACU
shLINC00922 sense obligo	CCGGATACTGGAAGTAGGGAATAAACTCGAGTTTATTCCCTACTTCCAGTATTTTTTG
shLINC00922 antisense obligo	AATTCAAAAAATACTGGAAGTAGGGAATAAACTCGAGTTTATTCCCTACTTCCAGTAT
siGDPD5 sense obligo	AGUUGAAUUCAUCAUAGUCAU
siGDPD5 antisense obligo	GACUAUGAUGAAUUCAACUGG
siNC sense obligo	UAUGAAUCAUGAGAAAUACAG
siNC antisense obligo	GUGAAUCCUAUUAUACUCUUU
shNC sense obligo	CCGGATACGAGTTTATTCCCTACTTCCAGTGGAAGTAGGCTAACTGAATAATTTTTTG
shNC antisense obligo	AATTCTGGAAGATAAACTCGAGTTTATTCCAAAAAATACCTACTAGGGATTCCAGTAT

Both parental and DDP-resistant GC cells were maintained in a 6-well plate at 37°C with 5% CO_2_. The transfection on these cells was performed with Lipofectamine 2000 reagent (11668–019, Invitrogen, Carlsbad, CA, USA) at 37°C when all cells reached the confluence of approximately 80%. 48 hours later, all cells were harvested for studies later.

### Dual-luciferase reporter assay

Sequence of LINC00922 containing the binding sites of miR-874-3p and that of GDPD5 containing the target sites of miR-874-3p were pre-inserted into the pMiRGLO dual-luciferase miRNA target expression vectors (E1330, Promega, Madison, WI, USA), and then the luciferase reporter plasmids of wild-type LINC00922 and GDPD5 (LINC00922-WT, GDPD5-WT) were constructed. In addition, the mutagenesis on the sequences was performed successfully with a site-directed mutagenesis kit (#200521, Agilent, Inc., Santa Clara, CA, USA) and luciferase reporter plasmids of mutated LINC00922 and GDPD5 (LINC00922-MUT, GDPD5-MUT) were made.

Both AGS/DDP and HGC-27/DDP cells at a density of 3 × 10^5^ cells/well were cultured within a 48-well plate and transfected with miR-874-3p mimic or mimic control and the luciferase reporter plasmids with Lipofectamine 2000 reagent at room temperature for 48 hours. Then, all cells were transferred to dual-luciferase reporter system for luciferase activity detection with Dual-Luciferase® Reporter Assay System (E1910, Promega, USA) according to the following procedures: all cells were first added with luciferase assay buffer II and incubated for 3 seconds, and the firefly luciferase activity was measured with SPECTROstar Nano microplate reader (BMG Labtech, Hopkinton, MA, USA) for 10 seconds. Subsequently, cells were injected with Stop & Glo buffer and additionally incubated for another 3 seconds. The *Renilla* luciferase activity was finally measured using the same microplate reader for another 10 seconds, the result of which was then used for normalizing the firefly luciferase activity. The luciferase assay buffer II and Stop & Glo buffer were provided from the kit and all procedures with the buffers were conducted in accordance with both the protocols of the manufacturer and a previous study [22]. The sequences used here were listed as follows: LINC00922-WT: 5’-CAGACAAGGCCAAGGCAAUCCCAGGGCAU-3’; LINC00922-MUT: 5’-CAGACAAGGCCAAGGCAAUCCCGAGCCAU-3’; GDPD5-WT: 5’-CUGCUGAUCUCCCUGCAGGGCAC-3’; GDPD5-MUT: 5’-CUGCUGAUCUCCCUGCACUGCAC-3’.

### Cell Counting Kit-8 (CCK-8) assay

5 × 10^3^ cells/well parental and DDP-resistant AGS and HGC-27 cells were firstly received transfection, and then treated with different concentrations of DDP (0, 1.25, 2.5, 5, 10, 20, 40, 80, and 160 μmol/L) for 48 hours [23]. 10 μL CCK-8 reagent (C0037, Beyotime Biotech, China) with serum-free medium was added into each well for viability detection at 37°C for another 4 hours. The optical density (OD) value at a wavelength of 450 nm was recorded with an Infinite® 200 PRO microplate reader (TECAN, Zürich, Switzerland). Cell viability was calculated using the following formula: cell viability (%) = [OD (dosage) - OD (blank)]/[OD (no dosage) - OD (blank)] × 100%, where OD (dosage) represented the OD value of the wells containing transfected and treated cells and CCK-8 reagent, OD (blank) symbolized that of the wells with only the medium and CCK-8 reagent, and OD (no dosage) was that of the wells, which were added with cells without any treatment, as described in a previous study [24]. Furthermore, the cell viability (%) was plotted against drug concentration to determine the IC_50_ value, that is, the drug concentration at which 50% of the cells were viable relative to control cells, and the estimated error was based on the average of 3 independent trials, which was calculated in line with prior discussion [25].

### Flow cytometric assay

1 × 10^6^ transfected parental and DDP-resistant AGS and HGC-27 cells were collected and transferred to 1.5 mL reaction tubes, followed by being centrifuged using Sorvall™ X4 Pro centrifuge (75009820, ThermoFisher Scientific, USA) at 1000 × *g* at 4°C for 5 minutes. The supernatant was removed, and 195 μL Annexin V-FITC binding buffer provided by the kit (C1062L, Beyotime Biotech, China) was added into cells. Then, all cells were gently resuspended and stained with 5 μL Annexin V-FITC and 10 μL propidium iodide (PI) working solutions. All cells were allowed to be incubated at room temperature for 15 minutes and protected from light. The staining buffer was abandoned via centrifugation at 500 × *g* at 4°C for 5 minutes, and cells were resuspended in 100 μL PBS. All samples were detected within FlowSight® flow cytometer (Luminex Corporation, Austin, TX, USA) and data were analyzed using Kaluza C Analysis Software (v. 3.1, Beckman Coulter, Indianapolis, IN, USA) as guided by the protocols of the manufacturers.

### Scratch assay

1 × 10^5^ transfected parental and DDP-resistant AGS and HGC-27 cells were first detached using Trypsin (T9935, Sigma-Aldrich, USA) and maintained within a 6-well plate to obtain complete confluence. Man-made scratches of a similar size were made on the monolayers of cells using a 10 μL sterile pipette tip in one direction once cells became entirely confluent.

After scratches, the monolayers of cells were washed gently to remove the detached cells. All remaining cells were incubated within serum-free medium, followed by being washed with PBS. Images of migrated cells were captured at 0 and 48 hours using an inverted optical microscope (IX71, Olympus, Tokyo, Japan) under × 100 magnification, and the migration rate was calculated   [26].

### Transwell assay

Prior to the Transwell assay, 10 mL Matrigel (M8370, Solarbio Lifesciences, China) was thawed at 4°C overnight, and diluted with chilled non-serum growth medium. Transwell chambers with 8-μm pore (CLS-3422, Sigma-Aldrich, USA) were coated with 50 μL pre-thawed Matrigel and placed on the 24-well plates that were placed in a humidified incubator at 37°C to allow gelling. Then, after being detached with Trypsin/Ethylenediaminetetraacetic acid (EDTA) (T3924, Sigma-Aldrich, USA), 1 × 10^5^ cells/well transfected parental and DDP-resistant AGS and HGC-27 cells were transferred to the upper Transwell chamber with 200 μL non-serum medium at 37°C with 5% CO_2_, and 700 μL complete medium was added into the corresponding lower chamber as the chemoattractant.

48 hours later, the excessive Matrigel was removed, while the lower Transwell chamber was fixed in 4% paraformaldehyde (P0099, Beyotime Biotech, China) at room temperature for 15 minutes and stained using 0.1% Giemsa (C0133, Beyotime Biotech, China) for 20 minutes. Invaded cells were photographed in five picked fields in a random manner under an inverted optical microscope at a magnification of × 250.

### Animal xenograft model construction

Animal xenograft model was constructed as illustrated in a prior study [27]. Male BALB/c nude mice (5-week-old, 20–25 g, n = 24) were preordered from Charles-River Laboratories (Wilmington, MA, USA) and housed in the specific non-pathogen cages with the conditions: light (12-hour cycle for day/night), temperature (22 ± 1°C), and humidity (55 ± 5%). Before the construction of animal xenograft model, all mice were freely accessed to the experimental chow and sterile tap water, and HGC-27/DDP cells were transfected with the shLINC00922 and its NC (shNC) which were synthesized and bought from GenePharma as well.

Then, we assigned all mice into the following groups: shNC, shLINC00922, shNC+DDP and shLINC00922+ DDP groups (n = 6 for each group). The treatment was as follows: all mice were received subcutaneous injection on the dorsal area with 2 × 10^6^ HGC-27/DDP cells, which have been transfected with shNC or shLINC00922. Then, all mice in shNC and shLINC00922 groups were further intraperitoneally injected with equivalent volume of saline, while those in shNC + DDP and shLINC00922+ DDP groups were additionally intraperitoneally injected with DDP (5 mg/kg) every 2 days [28]. The tumor volume was calculated every week for 4 weeks in total with the formula: tumor volume (mm^3^) = 0.5 × D × d^2^ (where D and d symbolized the longest and shortest diameters, respectively). Four weeks later, all mice were sacrificed via inhalation of 5% isoflurane (792632, Sigma-Aldrich, USA) and cervical dislocation, and the tumors were excised and weighed.

### RNA isolation and quantitative real-time polymerase chain reaction (qRT-PCR)

Total RNA in tissues (both drug-resistant and drug-sensitive), cells (treated or transfected GC cells and GES-1) and the tumor samples was isolated via TriZol (15,596–018, Invitrogen, USA). All miRNAs were extracted with an miRNA isolation kit (K1570-01, Invitrogen, USA) as instructed by the manufacturer. All RNA samples were preserved at −80°C, followed by the measurement on the concentration within an xMark microplate absorbance spectrophotometer (1681150, Bio-Rad, Hercules, CA, USA). All procedures within PCR were performed by a One-Step RT-PCR kit (D7268M, Beyotime Biotech, China) and operated in CFX96 Touch real-time PCR system (Bio-Rad, USA) under the following conditions: 94°C for 2 minutes, 94°C for 20 seconds, and 60°C for 34 seconds of 40 repeated cycles. Relative expressions were quantified via the 2^−ΔΔCT^ method with GAPDH and U6 as the housekeeping genes [29]. Primer sequences were referred in Table 2.

**Table 2.** Primers for qRT-PCR

Gene	Forward Primers (5’->-3’)	Reverse Primers (5’->-3’)
miR-874-3p	CCGAGTCGTATCCAGTGCAA	GTCGTATCCAGTGCGTGTCG
LINC00922	TCATCTAAGACTGGACCTTT	CAGTCACTGCTATGTGATTC
USP43	GGCATTACACAGCCTACT	GTAAGAGCCAGTGATCAGAC
GDPD5	GTGACCATTCTCTGTACCTT	GTAGAGGTTCACACTCAGGT
MYRF	GTAGACACTGATGGCTCTTT	CAAGGAGAGAGGCTGACT
PARP14	GGAATTAGCAGAGATGTGAT	GATTCTGGATCCTCTCAATC
SATB2	GATTAAAGTGGAAAGAGTGG	GCTGGTAGATATCTGGAGAG
RASGEF1A	CGTTAAGGACATCTACTTCC	ATGAACTCATGGATCTGTCT
RNF43	ACAGGAAAACTGAATCTCAC	GTTCTTGGTAAGATCGAGAG
KCNC3	AGACAAGGTGGAGTTTCTTA	GTTCTTGAAGTAGGTGTGGT
CDH11	AGTGAAAGCTAAAGATCCAG	TCTCTATCCAGAGGTTTTGT
U6	CTCGCTTCGGCAGCACATATACT	ACGCTTCACGAATTTGCGTGTC
GAPDH	TTTTTGGTTTTAGGGTTAGTTAGTA	AAAACCTCCTATAATATCCCTCCTC

### Western blot

Relative protein expressions were measured with Western blot, and the processes mentioned here were confirmed in a prior study [30]. Total protein in treated or transfected cells and the tumor samples was firstly lysed using Radioimmunoprecipitation (RIPA) buffer (P0013C, Beyotime Biotech, China). The quantification on the concentration of the protein was conducted with the bicinchoninic acid (BCA) method via a BCA protein kit (P0012S, Beyotime Biotech, China). 20 μL of total protein lysates were electrophoresed with sodium dodecyl sulfate-polyacrylamide gel electrophoresis (SDS-PAGE) (P0012A, Beyotime Biotech, China), and transferred into polyvinylidene fluoride (PVDF) membrane (FFP33, Beyotime Biotech, China). Then, the membrane was blocked using 5% nonfat milk at room temperature for 2 hours, and then incubated with primary antibodies at 4°C overnight and secondary antibodies at room temperature for 1 hour. For subsequent procedures, the membrane was washed using tris-buffer saline tween (TBST, T1085, Solarbio Lifesciences, China) three times, and visualized in enhanced chemiluminescence Western blotting substrate (PE0010, Solarbio Lifesciences, China) within iBright FL1500 Imaging System (A44115, Invitrogen, USA). The gray values were calculated using ImageJ (v. 5.0, Bio-Rad, USA). The primary antibodies used in this research were those against GDPD5 (1:2000, ABIN5689971, Antibodies-online GmbH, Aachen, Germany, https://www.antibodies-online.com) and internal control GAPDH (1:5000, ABIN2857072, Antibodies-online GmbH, Germany), while the secondary antibody adopted here was horseradish peroxidase (HRP)-conjugated goat anti-rabbit IgG antibody (1:2000, ab205718, Abcam, Cambridge, UK).

## Statistical analyses

Statistical analyses were performed with SPSS software (SPSS, Chicago, IL, USA), in which all data, the indication of three independent tests, were expressed as mean ± standard deviation (SD). Statistical significance was determined with one-way analysis of variance (ANOVA) followed by Bonferroni post hoc test and paired *t* test, which defined as *p-*value < 0.05.

## Results

Chemotherapy resistance severely limits the therapeutic effect of gastric cancer patients. The purpose of this study was to investigate the role and mechanism of miR-874-3p in regulating drug resistance to GC cells. In this study, we found that miR-874-3p was lowly expressed in DDP-resistant GC tissues and cells, and miR-874-3p inhibited the migration and invasion, but promoted the apoptosis and Cisplatin sensitivity via being sponged by LINC00922 to target GDPD5 in GC cells. This provided the evidence regarding the roles of miR-874-3p in both the progression of and drug resistance to GC cells altogether.

## MiR-874-3p was lower-expressed in drug-resistant GC tissues and cells

At the beginning of our study, miR-874-3p expression was confirmed in GC tissues and cells. In accordance with the results, miR-874-3p expression was firstly found to be decreased in drug-resistant GC tissues (*p* < 0.001; Figure 1(a)). Meanwhile, miR-874-3p expression was observed to be downregulated in GC cells compared with that in GES-1 cells, with lower miR-874-3p expression being further evidenced in DDP-resistant GC cells (AGS/DDP and HGC-27/DDP) (*p* < 0.001; Figure 1(b)).

## MiR-874-3p upregulation repressed the cell viability, migration and invasion but enhanced apoptosis and sensitivity in DDP-resistant or parental GC cells

We then set out to unveil the possible effect of miR-874-3p on DDP-resistant GC cells, so we first transfected miR-874-3p mimic and its control (mimic control) into DDP-resistant or parental GC cells, and upregulated miR-874-3p expression in DDP resistant or parental GC cells suggested the successful transfection (*p* < 0.001; Figure 1(c,d)). Besides, in accordance with the results from CCK-8 and flow cytometric assays, it was uncovered that upregulation of miR-874-3p in DDP-resistant or parental GC cell resulted in the decreased cell viability and IC_50_ value yet a promotive effect on cell apoptosis (*p* < 0.05; Figure 1(e–h)). Scratch and Transwell assays were applied to evaluate the effects of miR-874-3p on the migration and invasion of DDP-resistant or parental GC cells. In line with the results, we found that the upregulation of miR-874-3p was associated with the decrease on the migration and invasion of DDP-resistant or parental GC cells (*p* < 0.001; Figure 2(a–d)). It could be concluded from the results above that miR-874-3p upregulation elicited a suppression on the viability, migration and invasion as well as a promotion on sensitivity to DDP and apoptosis in DDP-resistant or parental GC cells.

## LINC00922 was the upstream lncRNA for miR-874-3p

LncBase Predicted V2 (http://carolina.imis.athena-innovation.gr/diana_tools) was then employed to predict the upstream lncRNA for miR-874-3p, among which LINC00922 aroused our interest. One prior study has unveiled the oncogenic role of LINC00922 and its potential role in doxorubicin-resistant osteosarcoma[31]. However, whether LINC00922 also exerted its effects on DDP-resistant GC cells was inadequately discussed. Thus, we speculated that LINC00922 could be the upstream lncRNA for miR-874-3p, as indicated by the potential-binding sites between miR-874-3p and LINC00922 (which was available from LncBase and displayed in Figure 3(a)). The results from the dual-luciferase reporter assay confirmed that LINC00922 was indeed the upstream lncRNA for miR-874-3p, where the luciferase activities in DDP-resistant GC cell transfected with LINC00922-WT and miR-874-3p mimic were decreased in comparison with those transfected with LINC00922-WT and miR-874-3p mimic control (*p* < 0.01; Figure 3(b,c)), whereas no significant changes were found on the luciferase activity in DDP-resistant GC cells transfected with LINC00922-MUT and miR-874-3p mimic or mimic control. Collectively, we here demonstrated that LINC00922 was the upstream lncRNA for miR-874-3p.

## Downregulated miR-874-3p eliminated the effects of LINC00922 silencing on miR-874-3p expression, cell viability, apoptosis, migration and invasion of DDP-resistant GC cells

To affirm the interaction between miR-874-3p and LINC00922 on DDP-resistant GC cells, we transfected both siLINC00922 and miR-874-3p inhibitor into DDP-resistant GC cells, in which we confirmed the successful transfection, as evidenced by the reduction on both LINC00922 and miR-874-3p expression following the transfection (*p < *0.001; Figure 3(d–g)). We also measured miR-874-3p expression in DDP-resistant cells following the downregulation on miR-874-3p and the silence of LINC00922. When it comes to the results, miR-874-3p expression was upregulated after silencing LINC00922, whereas miR-874-3p inhibitor repressed the expression of miR-874-3p (*p* < 0.001; Figure 3(h–i)). We additionally found that miR-874-3p downregulation reversed the effects of LINC00922 silencing on miR-874-3p expression in DDP-resistant GC cells (*p* < 0.001; Figure 3(h–i)). LINC00922 silencing inhibited the expression of LINC00922, while miR-874-3p downregulation had no effect on the LINC00922 expression (*p* < 0.001; Figure 3(j–k)).

We also determined the interaction between miR-874-3p and LINC00922 on the viability, apoptosis, migration and invasion of DDP-resistant GC cells. According to the results from CCK-8 assay, we found the inhibitor of miR-874-3p was related to increase on cell viability, with an increased IC_50_ value (*p* < 0.05; Figure 4(a,b)), while LINC00922 silencing did conversely, with a decreased IC_50_ value (*p* < 0.05; Figure 4(a,b)). Furthermore, the downregulation on miR-874-3p countervailed the effects of LINC00922 silencing on both cell viability and IC_50_ value in DDP-resistant GC cells (*p* < 0.05; Figure 4(a,b)). In accordance with the results from flow cytometry, we found that LINC00922 silencing enhanced apoptosis whereas downregulation of miR-874-3p suppressed the apoptosis of DDP-resistant GC cells. It was further unveiled that miR-874-3p downregulation eliminated the effects of LINC00922 silencing on the apoptosis in DDP-resistant GC cells (*p* < 0.001; Figure 4(c,d)). Besides, LINC00922 silencing was discovered to repress both the migration and invasion of DDP-resistant GC cells, whereas miR-874-3p downregulation elicited contrary results (*p* < 0.001; Figure 5(a–d)). Furthermore, it was demonstrated that miR-874-3p downregulation could reverse the effects of LINC00922 silencing on the migration and invasion of DDP-resistant GC cells (*p* < 0.001; Figure 5(a–d)). To sum up, the downregulating miR-874-3p could eliminate the effects of LINC00922 silencing on the viability, apoptosis, migration and invasion of DDP-resistant GC cells.

## GDPD5 was the downstream target gene of miR-874-3p

Additionally, the downstream target gene of miR-874-3p was predicted and confirmed as well, and several databases, including TargetScan, StarBase and TCGA-STAD, were searched, the results of which were finally intersected using a Venn diagram, where nine common genes were sorted and displayed in Figure 6(a). Then, we measured their expressions in DDP-resistant GC cells following the downregulation of miR-874-3p, and the expressions of all these genes were upregulated following the downregulation of miR-874-3p, among which the expression of GDPD5 was the highest (*p* < 0.05; Figure 6(b,c)). Besides, we assumed that GDPD5 was the downstream target gene of miR-874-3p as well, as GDPD5 was the target gene of miRNA and associated with the DDP-associated resistance [32]. The assumption was further proved by the data from TargetScan and the results of dual-luciferase reporter assay, as indicated by the discovery that the luciferase activity in DDP-resistant GC cells that has been transfected with GDPD5-WT and miR-874-3p mimic was decreased as compared to that in cells transfected with GDPD5-WT and miR-874-3p mimic control (*p* < 0.001; Figure 6(d–f)), in addition to the results where no significant changes were found on the luciferase activity in DDP-resistant GC cells, which have been transfected with GDPD5-MUT and miR-874-3p mimic or mimic control.

## MiR-874-3p downregulation eliminated the effects of LINC00922 silencing on GDPD5 expression in DDP-resistant GC cell

**Figure 7.**
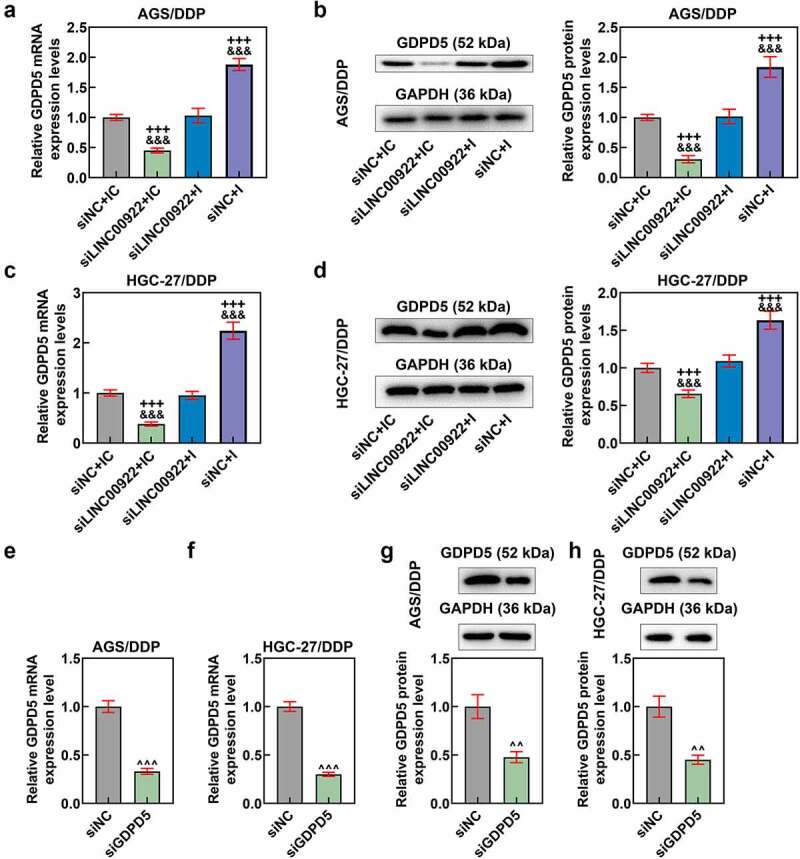
MiR-874-3p downregulation eliminated the effects of LINC00922 silencing on GDPD5 in DDP-resistant GC cells, the expression of which was also decreased via transfection. (a-d) The effects of LINC00922 silencing and miR-874-3p downregulation on GDPD5 expression of DDP-resistant GC cell AGS/DDP (a-b) and HGC-27/DDP (c-d) were determined by qRT-PCR and Western blot. GAPDH was the housekeeping gene. (e-h) GDPD5 expression was measured again in DDP-resistant GC cell AGS/DDP and HGC-27/DDP following the transfection of siGDPD5, as evidenced by qRT-PCR (e-f) and Western blot (g-h). GAPDH was the housekeeping gene. All data were expressed as mean ± standard deviation (SD), which was indicative of three independent tests. ^+++^*p < *0.001, vs. siNC+IC; ^&&&^*p < *0.001, vs. siLINC00922 + I; ^^^^^*p < *0.001, vs. siNC.

We also determined the interaction between miR-874-3p and LINC00922 on GDPD5 expression in DDP-resistant GC cells. In light of the results, the silence of LINC00922 was related to the downregulation of GDPD5 in DDP-resistant GC cells, whilst miR-874-3p downregulation caused the upregulation of GDPD5 (*p* < 0.001; Figure 7(a–d)). We additionally identified that miR-874-3p downregulation countervailed the effects of LINC00922 silencing on GDPD5 expression in DDP-resistant GC cells (*p* < 0.001; Figure 7(a–d)).

## GDPD5 silencing diminished the effects of downregulating miR-874-3p on GDPD5 expression and cell viability of DDP-resistant GC cells

**Figure 8.**
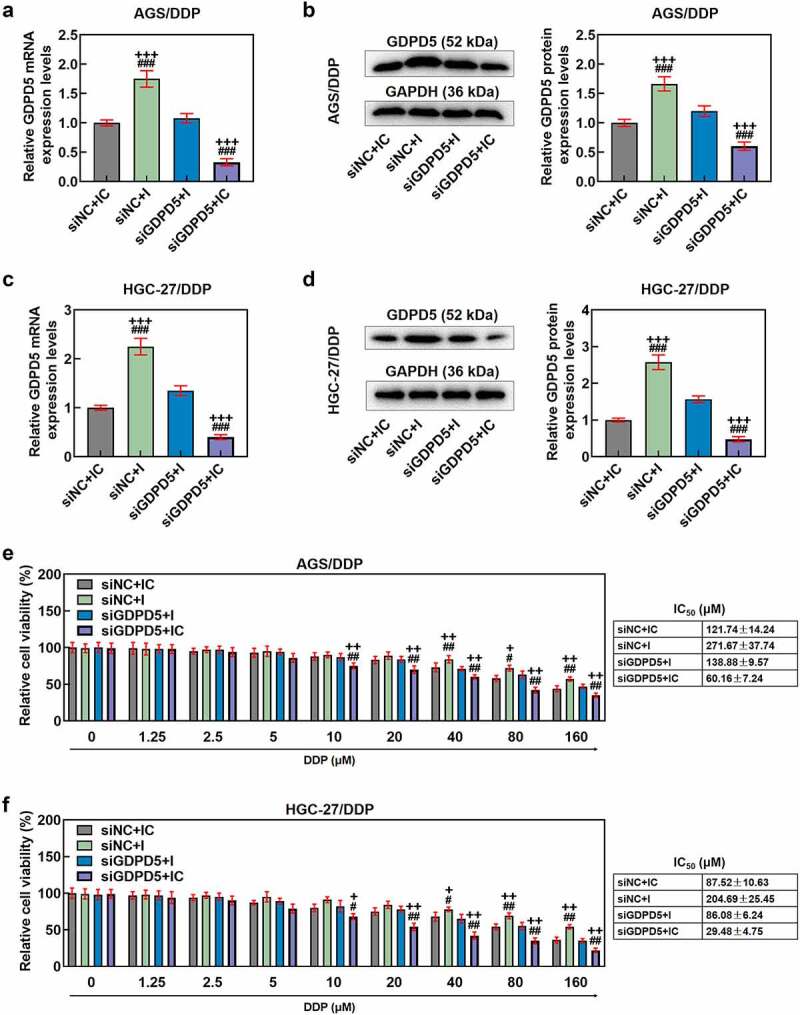
GDPD5 silencing diminished the effects of downregulated miR-874-3p on GDPD5 expression and cell viability of DDP-resistant GC cells. (a-d) The effects of GDPD5 silencing and miR-874-3p downregulation on GDPD5 expression in DDP-resistant GC cell AGS/DDP (a-b) and HGC-27/DDP (c-d) were unveiled as confirmation of qRT-PCR and Western blot. GAPDH was used as the internal control. (e-f) The effects of GDPD5 silencing and miR-874-3p downregulation on the cell viability in DDP-resistant GC cell AGS/DDP (e) and HGC-27/DDP (f) were suggested by CCK-8 assay. All data were expressed as mean ± standard deviation (SD), which was indicative of three independent tests. ^+^*p < *0.05, ^++^*p < *0.01, ^+++^*p < *0.001, vs. siNC+IC; ^#^*p < *0.05, ^##^*p < *0.01, ^###^*p < *0.001, vs. siGDPD5 + I.

In this phase, the interaction between GDPD5 and miR-874-3p in DDP-resistant GC cells was analyzed. We first transfected DDP-resistant GC cells with siGDPD5, the success of which was evidenced by the decrease on GDPD5 expression in DDP-resistant cells (*p* < 0.001; Figure 7(e–h)). Meanwhile, we ascertained that downregulation of miR-874-3p resulted in increased GDPD5 expression and IC_50_ value andcell viability (*p < *0.05; Figure 8(a–f)). However, GDPD5 silencing not only elicited opposite results but also diminished the effects of miR-874-3p downregulation on DDP-resistant GC cells (*p < *0.05; Figure 8(a–f)).

## GDPD5 silencing diminished the effects of downregulated miR-874-3p on the migration and invasion of DDP-resistant GC cells

**Figure 9.**
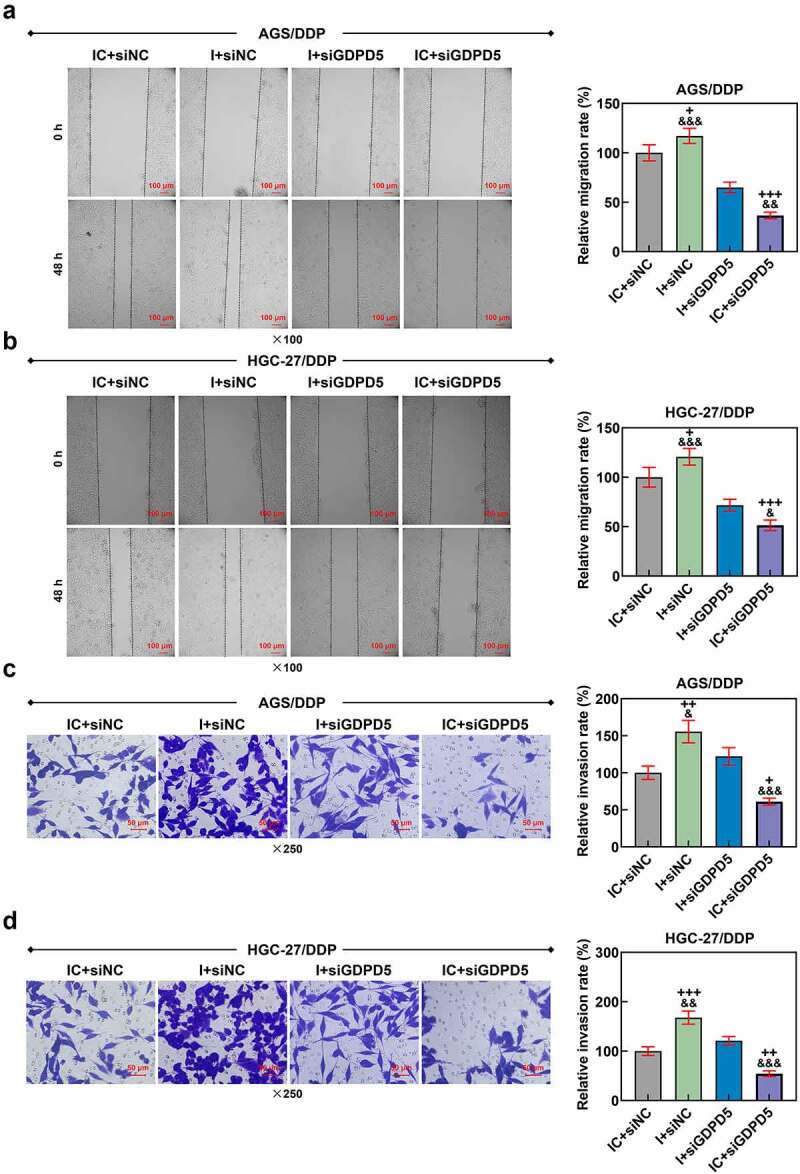
GDPD5 silencing diminished the effects of downregulated miR-874-3p on the migration and invasion of DDP-resistant GC cells. (a-b) The effects of GDPD5 silencing and miR-874-3p downregulation on the migration of DDP-resistant GC cells AGS/DDP (a) and HGC-27/DDP (b) were confirmed with Scratch assay at 0 and 48 hours, under × 100 magnification (Scale bar = 100 μm). (c-d) The effects of GDPD5 silencing and miR-874-3p downregulation on the invasion of DDP-resistant GC cells AGS (c) and HGC-27 (d) at 48 hours were detected with Transwell assay (× 250 magnification; Scale bar = 50 μm). All data were expressed as mean ± standard deviation (SD), which was indicative of three independent tests. ^+^*p < *0.05, ^++^*p < *0.01, ^+++^*p < *0.001, vs. IC+siNC; ^&^*p < *0.05, ^&&^*p < *0.01, ^&&&^*p < *0.001, vs. I+ siGDPD5.

In addition, we detected the effect of GDPD5 silencing on the migration and invasion of DDP-resistant GC cells. The result showed that downregulation of miR-874-3p promoted the migration and invasion of DDP-resistant GC cells, while GDPD5 silencing inhibited the migration and invasion of DDP-resistant GC cells and diminished the effects of miR-874-3p downregulation on DDP-resistant GC cells (*p < *0.05; Figure 9(a–d)).

## LINC00922 silencing enhanced the effects of DDP on repressing growth in tumors

**Figure 10.**
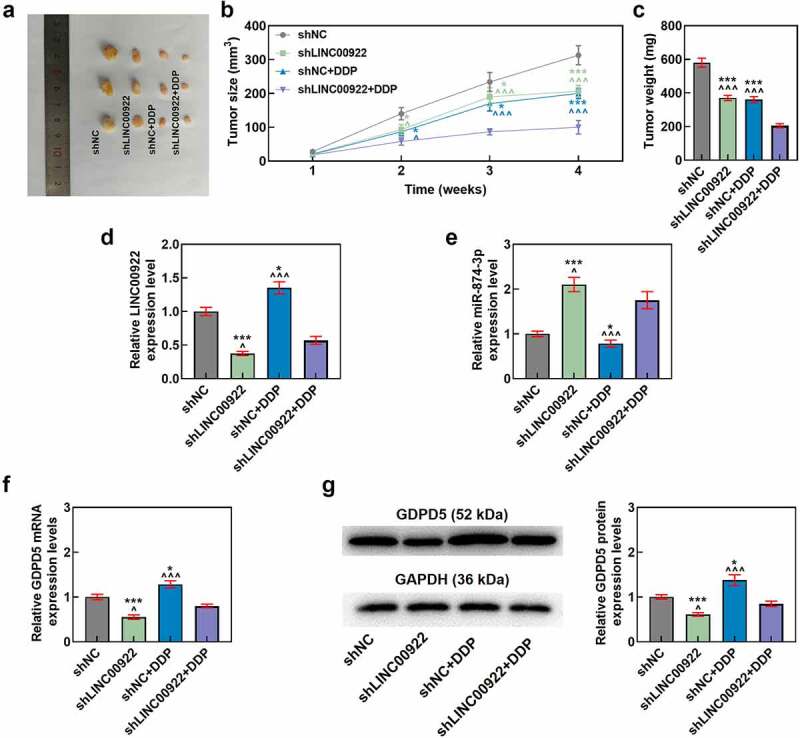
LINC00922 silencing further repressed tumor growth and LINC00922 and GDPD5 yet enhanced miR-874-3p in DDP-treated tumors. (a-c) The effects of LINC00922 silencing on DDP-treated tumor growth *in vivo* were displayed, along with the measurement on both tumor size (b) and tumor weight (c). (d-g) The effects of LINC00922 silencing on LINC00922 (d), miR-874-3p (e) and GDPD5 (f-g) expression in DDP-treated tumors were confirmed by qRT-PCR and Western blot. GAPDH (for LINC00922 and GDPD5) and U6 (for miR-874-3p) were used as the internal controls. All data were expressed as mean ± standard deviation (SD), which was indicative of three independent tests. ^^^*p < *0.05, ^^^^^*p < *0.001, vs. shNC; *p < 0.05, ***p < 0.001, vs. shLINC00922+ DDP. shRNA: short hairpin RNA.

Finally, we set out to assess the effects of LINC00922, miR-874-3p and GDPD5 in DDP-treated tumors *in vivo*. In line with the results, both LINC00922 silencing and DDP treatment repressed tumor growth and weight, while LINC00922 silencing further suppressed DDP-treated tumor growth *in vivo* (*p* < 0.05; Figure 10(a–c)).

## LINC00922 silencing offset the effects of DDP on the expressions of LINC00922, miR-874-3p and GDPD5 in tumors

As for LINC00922, miR-874-3p, and GDPD5 expressions *in vivo*, we found that LINC00922 silencing repressed the expressions of LINC00922 and GDPD5 yet enhanced that of miR-874-3p, while DDP treatment did the opposite, that is, upregulated the expressions of LINC00922 and GDPD5 but downregulated that of miR-874-3p (*p* < 0.05; Figure 10(d–g)). Furthermore, LINC00922 silencing was discovered to offset the effects of DDP on enhancing the expressions of LINC00922 and GDPD5 yet reducing that of miR-874-3p in tumors (*p* < 0.05; Figure 10(d–g)).

## Discussion

Increasing evidence has unveiled the different roles of miR-874-3p in both the progression and drug resistance in GC [14,16]. We, hereby, not only reconfirmed the effects of miR-874-3p on both the progression and drug resistance in GC, but also provided sufficient evidence concerning the upstream lncRNA and downstream target gene for miR-874-3p. Specially, we first confirmed the downregulation of miR-874-3p in drug-resistant GC tissues and DDP-resistant GC cells, and then found that upregulation of miR-874-3p contributed to the increase in both sensitivity and apoptosis as well as the decrease in migration and invasion of both DDP-treated and untreated GC cells. Further results additionally demonstrated that lncRNA LINC00922 and GDPD5 were the upstream lncRNA and the downstream gene for miR-874-3p, and the interactions among LINC00922, miR-874-3p and GDPD5 have been proved as well. Moreover, it was shown that DDP-induced reduced miR-874-3p expression was eliminated by LINC00922 *in vivo*, and silencing LINC00922 enhanced chemosensitivity in GC cells and inhibited the tumor growth. From these results above, it could thus be concluded that miR-874-3p was lowly expressed in DDP-resistant GC cell, and its upregulation could enhance the apoptosis and DDP chemosensitivity yet repress the progression of GC, which were achieved via being sponged by LINC00922 and targeting the downstream target gene GDPD5, providing the evidence regarding the roles of miR-874-3p in both the progression and drug resistance in GC altogether.

GC has been documented to be originated from the accumulation of a series of epigenetic alternations in both oncogenes and tumor suppressor genes, resulting in the dysregulation on multiple signaling pathways, which further elicits a disruption on the cell cycle and the balance between cell proliferation and death. The proliferation is one of those major cell events related to the initiation of tumors, while apoptosis is defined as a programmed cell death process, which is a key part of the innate tumor-suppression mechanism [33–36]. In addition, metastasis, an important element of GC, leads to high mortality rate and poor prognosis in patients, which is implicated in the release of circulating tumor cells (CTCs) or other major blood biomarkers into the circulating blood stream by the tumors and also is underlined to accelerate the progression of GCs. As such, metastasis is perceived as one of the two major causes of the poor prognosis of GC patients, with the other one being recurrence [37–40]. Prior reports have detected the abnormal pattern of miRNAs and affirmed the participation of miRNAs in the tumorigenesis, progression, and metastasis of GC, as well as the discoveries, suggesting that miR-874-3p unleashed the suppressive effects on the growth, migration, invasion, and tumorigenicity as well as the promotive effects on the apoptosis of GC cell, and brought about the downregulation of migration- and invasion-associated factors Membrane type 1-matrix metalloproteinase (MT1-MMP), matrix metalloproteinase-2 (MMP-2), and MMP-9 as well as the regulation on apoptosis-related participators, that is, the upregulation of caspase-3 and Bcl-2 associated X (Bax) and the suppression of B-cell lymphoma-2 (Bcl-2) [14,41]. In our current study, likewise, we also discovered that the upregulation of miR-874-3p was associated with the suppression on the viability, migration and invasion as well as the promotion on the apoptosis in GC cells with or without DDP, whereas downregulation of miR-874-3p did conversely, which provided another evidence with respect to the inhibitory role of miR-874-3p in GC.

GC, along with many other malignancies, like pancreatic cancer and malignant melanoma, for instance, shows a particularly high level of intrinsic drug resistance to the chemotherapeutic agents, which includes the classical multidrug resistance phenomenon [42]. As a major failure in chemotherapy for cancer treatment, multidrug resistance accounts for more than 90% of total deaths in cancer patients who have received conventional chemotherapies or novel target medications [43,44]. DDP, a platinum-based anti-tumor drug, has been indicated to be used as a therapeutic agent for chemotherapy in a wide range of malignancies worldwide [45,46]. Currently, DDP remains as the primary chemotherapeutic drug for treating patients with GC, those who have been diagnosed with an advanced stage in particular [47]. However, the tumor microenvironment (TME), which protects tumor cells against treatment can be also contributory to the genetic changes of tumor cells, such as increased drug efflux or enhanced apoptosis evasion and drug resistance. For this reason, the intrinsic or acquired resistance on cancer cells has left a severe limitation on the clinical use of DDP [48,49]. Thus, it’s of great significance and urgency to figure out a viable therapeutic method to overcome or diminish the DDP-induced drug resistance in cancer cells [50]. Prior discovery has underlined that upregulation of miR-874-3p could increase the sensitivity of GC cell to chemotherapeutic drugs *in vitro*, including 5-fluorouracil (5-Fu) and DDP [16]. In our study, we also proved that the upregulation of miR-874-3p enhanced the sensitivity to DDP in GC cell, while the downregulation of miR-874-3p did conversely, further confirming the significance of miR-874-3p in drug sensitivity of GC.

In order to further confirm the mechanism via which miR-874-3p elicited its effects on GC cells, we predicted both the upstream lncRNA and the downstream target gene for miR-874-3p, among which LINC00922 and GDPD5 were sorted and identified. As a member in the lncRNA family, LINC00922 has been unveiled to act as an oncogene in various cancers, osteosarcoma for instance [31]. In addition, it has been documented that LINC00922 led to the acceleration of lung cancer via the sponge of miR-204 [51]. The discovery made us wonder whether miR-874-3p was additionally sponged by LINC00922 and whether LINC00922 was also implicated in the development and progression of GC, as none of these have been properly addressed so far. As displayed in our results, we first provided evidence that miR-874-3p was sponged by LINC00922, and the downregulation of miR-874-3p eliminated the effects of LINC00922 silencing on both the progression and drug resistance in GC cells. Further experiments *in vivo* supported the results in regard to the interaction between these two, as shown by the discovery that LINC00922 silencing eliminated the effects of DDP on suppressing miR-874-3p expression *in vivo*. These results hereby provided evidence concerning the role of LINC00922 on GC and further confirmed the interaction between miR-874-3p and LINC00922.

GDPD, as a family of enzymes that can hydrolyze glycerophosphodiesters into sn-glycerol-3-phosphate and the corresponding alcohols, is pivotal in a variety of physiological processes in the prokaryotes and eukaryotes [52]. Previous discoveries have also provided a great deal of evidence concerning the involvement of GDPD5, a member of the GDPD family, in the progression of tumors and the DDP resistance in tumors by acting as the target gene of miRNAs, as well as the discussion on the interaction between GDPD5 and miR-874-3p in colorectal cancer, where GDPD5 was identified as the target gene of miR-874-3p and overexpressed GDPD5 eliminated the effects of miR-874-3p triggered on the tumor inhibition and ferroptosis of colorectal cancer cells [32,53,54]. Nevertheless, the role of GDPD5 in the mechanism via which miR-874-3p exerted its effects on GC needed to be additionally elucidated. We, in our study, reconfirmed GDPD5 as the downstream target gene of miR-874-3p, and found that GDPD5 silencing not only repressed the viability but also led to a reversion on the effects of miR-874-3p downregulation on GC cells.

However, there are some shortages in our current study we want to address here. For instance, we solely confirmed the expressions of miR-874-3p, LINC00922 and GDPD5 *in vivo*, but their specific effects *in vivo* were dearth of a further systemic elucidation. In addition, more experiments were required to further confirm the interaction between miR-874-3p and GDPD5 in GC cells.

## Conclusion

Taken together, we provide new evidence on the role of miR-874-3p in the progression and drug resistance in GC cells. To be specific, we confirmed that miR-874-3p, an miRNA that was not only sponged by LINC00922 but also targeted GDPD5, could suppress the migration and invasion yet enhance apoptosis and DDP resistance in GC cells. We hope that the results we proposed here can be contributory to providing both evidences concerning the implication of miR-874-3p or other miRNAs in GC and a potentially viable therapeutic method for GC in the future.

## Data Availability

The analyzed data sets generated during the study are available from the corresponding author on reasonable request.

## Highlights

- MiR-874-3p is low-expressed in drug-resistant GC tissue and cell.
- MiR-874-3p is sponged by LINC00922 and targets GDPD5 in GC.
- MiR-874-3p inhibits GC cell progression but enhances apoptosis and sensitivity.
